# Commentary: Group 3 innate lymphoid cells mediate early protective immunity against tuberculosis

**DOI:** 10.3389/fimmu.2020.01925

**Published:** 2020-08-27

**Authors:** Boning Zeng, Rui Xing, Changjiang Dong, Feiyue Xing

**Affiliations:** ^1^Department of Immunobiology, Institute of Tissue Transplantation and Immunology, Jinan University, Guangzhou, China; ^2^MOE Key Laboratory of Tumor Molecular Biology, Key Laboratory of Functional Protein Research of Guangdong, Higher Education Institutes, Jinan University, Guangzhou, China; ^3^MOE Key Laboratory of Protein Sciences, Tsinghua-Peking Center for Life Sciences, School of Life Sciences, Tsinghua University, Beijing, China; ^4^BioMedical Research Center, University of East Anglia, Norwich, United Kingdom

**Keywords:** group 3 innate lymphoid cell, pulmonary tuberculosis, mycobacterium tuberculosis, CXCR5, CXCL13, iBALT

Tuberculosis (TB), caused by *Mycobacterium tuberculosis* (*Mtb*), is a leading cause of mortality, surpassing any other single infectious disease. Two billion people are infected with *Mtb* worldwide with 1.7 million deaths each year (1). One of the most critical factors leading to the global epidemic of TB is lack of adequate and safe vaccines. Although the current only licensed TB vaccine, Bacille Calmette-Guérin (BCG), can prevent *Mtb* infection in infants and young children, it can only provide partial and inconsistent protection (0–70%) to populations (1, 2). Most studies showed that BCG vaccination protection against tuberculosis lasts for up to 10 years, waning with time (3). Moreover, mounting explosion of drug-resistant TB and TB/HIV co-infection poses a serious threat to treatment and further prevention of the diseases (4, 5). Over the past decade, a worldwide collaborative effort to develop a novel preventive and/or therapeutic TB vaccine is unfortunately disappointing. Although some encouraging progress has been made, developing a vaccine more effective than BCG, and a new therapeutic strategy for treatment, remains an ongoing challenge, especially due to lack of crucial biomarkers and poor understanding of the mechanistic processes involving innate and adaptive immunity in the protection against this disease (4, 6).

Innate immunity against TB is very crucial in the control of early *Mtb* infection especially by pulmonary immune responses, which is mainly carried out by alveolar innate immune cells, such as macrophages, dendritic cells, monocytes, and neutrophils. Adaptive immunity against TB is also executed by Th1 and Th17 cells with TNF-α, IFN-γ, IL-17, and IL-22. Both IL-17 and IL-22 can recruit neutrophils, and activate macrophages, IFN-γ-producing CD8^+^ T cells, γδT cells with perforin and granulysin as well as other T lymphocytes, including NKT and CD1-restricted αβT cells (7, 8). Additionally, B cells in *Mtb*-infected lungs are also able to combat *Mtb* especially through generation of specific or nonspecific antibodies in a T cell-dependent or independent manner. They present *Mtb* antigens to Th cells, such as Th1, Th17, and Tfh cells. The latter release IFN-α and IFN-β to function, facilitate neutrophil migration via regulating Th17 cells to produce IL-17, and polarize lung macrophages (9).

Recently, a ground-breaking study on group 3 innate lymphoid cells (ILC3s) was reported in *Nature* (10). Ardain et al. (10) reveal for the first time that ILC3s mediate an early protective role in immunity to *Mtb* infection in lungs through generation of IL-17 and IL-22 in a C-X-C motif chemokine ligand 13 (CXCL13)–C-X-C motif chemokine receptor 5 (CXCR5)-dependent manner with formation of inducible bronchus-associated lymphoid tissue (iBALT) structures, providing a novel insight into the mechanistic process of interaction between TB infection and innate immune cells (Figure 1).

**Figure 1:**
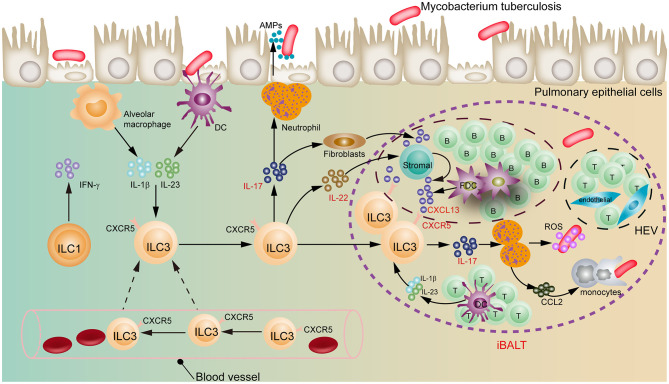
Regulatory role of ILC3s in early pulmonary tuberculosis. ILC3s move from blood to lung, and recruit immune cells through the IL-17 and IL-22 to form iBALT structures in a CXCL13-CXCR5-dependent manner, exerting an early protective effect on *Mtb* infection. DC, dendritic cells; FDC, follicular dendritic cells; HEV, high endothelial venules; iBALT, inducible bronchus-associated lymphoid tissues.

Recognized only in the past decade, ILCs have been currently classified into group 1 innate lymphoid cells (ILC1s), natural killer (NK) cells, group 2 innate lymphoid cells (ILC2s), ILC3s, lymphoid-tissue-inducer (LTi) cells, and regulatory innate lymphoid cells (ILCregs) on the basis of their development, phenotype, and function (11–13). ILC1s mainly produce the Th1-type cytokine IFN-γ. ILC2s secrete Th2-type cytokines, such as interleukin (IL)-4, IL-5, IL-9, and IL-13. ILC3s, including natural cytotoxicity receptor-negative (NCR^−^) and NCR^+^ ILC3s, primarily produce IL-17 and IL-22, like Th17 and Th22 cells. NK cells functionally represent the counterpart of the CD8^+^ cytotoxic T cells. Finally, ILCregs release both IL-10 and TGF-β to mirror the functions of Tregs (11, 14, 15). It is well-known that ILC3s exist mainly in intestinal mucosa, epithelial tissue, and skin, which are considered to function in tissue repair and mucosal homeostasis, whereas ILC2s are largely located in lung, skin, adipose tissue, and peripheral lymphatic tissues, which enable these tissues and organs to defend against helminth infection and allergen invasion.

The involvement of ILC3s in respiratory diseases is, to date, less well-defined. Inspiringly, Ardain et al. findings, that ILC3s but not ILC2s can protect lung tissue from *Mtb* early infection, make us regain recognition of novel actions of ILC3s in lungs. Importantly, they can initiate not only a rapid, non-specific response to extracellular microbes, but also a protection against intracellular specific pathogen *Mtb*, which is regarded as a crucial regulator in tissue homeostasis and inflammatory response (12). In this study, the researchers observed that the population of ILCs, including the ILC3s, was highly exhausted in *Mtb*-infected patients compared to healthy individuals. Intriguingly, the number of ILC1s and ILC3s, but not ILC2s, restored upon treatment, indicating that *Mtb* bacteremia can modulate the accumulation of ILCs. Using human and mouse models of *Mtb* infection, they found that the accumulation of ILC3s synchronizes with alveolar macrophages increased in the lungs to reinforce *Mtb* control. The authors also tracked the localization of ILCs in several animal models and discovered that in the mice with intact immune system ILC3s migrate from the blood to the lung, homing to infected lung tissue, and produce messenger molecules IL-17 and IL-22. These cytokines are critical inducers of CXCL13 to recruit immune cells to form iBALT-associated granuloma structures in a CXCL13-CXCR5-dependent manner (Figure 1). Additionally, ILC3s are also able to inhibit *Mtb* growth in a phagolysosomal fusion manner (16).

CXCL13, one of the members of the CXC-chemokine family, is usually expressed by stromal cells, follicular dendritic cells (FDCs) and CD11c^+^ DCs during *Mtb* infection (17). In fact, increasing evidence supports that a CXCL13-CXCR5 axis is associated with the formation of iBALT (17, 18). iBALT consists principally of separated B cell areas containing FDCs, T cell regions containing DCs, high endothelial venules (HEVs), specialized stromal cells as well as lymphatic vessels (18, 19). Consistent with these reports, Ardain et al. found that the level of CXCL13 was elevated in *Mtb*-infected mice and patients with TB. Furthermore, the expression of CXCR5, a receptor of CXCL13, on the surface of ILC3s was also upregulated, suggesting that the CXCL13-CXCR5 axis might be involved in the regulation of *Mtb* infection by ILC3s (10). Additionally, ILC3s appear highly heterogeneous. Phenotypic characteristics of NCR^−^ ILC3s include Lin^−^, CCR6^+^, NKp44^−^ (in humans), CD25^+^, CD117^+^, CD127^+^, and CD161^+^, but NCR^+^ ILC3s present NKp44^+^ and NKp30^+^, which is entirely discriminated from NCR^−^ ILC3s (8). Thus, it deserves to be further studied what is a regulatory role of each ILC3 subpopulation in TB infection. Noteworthily, in the lungs of immunodeficient mice without functional ILCs the number of macrophages was decreased with the poor immune control of TB, which demonstrates that macrophages can mediate especially activation of ILC3s through IL-1β.

On the other hand, intestinal CD103^+^CD11b^+^ DCs instruct the lung-specific trafficking of circulating IL-22^+^ ILC3s via boosting the lung-homing receptor CCR4 expression on IL-22^+^ ILC3s. The CCR4 is recruited by chemokine CCL17 or CCL20 generated by pulmonary epithelium cells (20). Ly6C^low^CD11b^+^ activated monocytes increases the frequency of IL-17^+^ ILC3s in the lungs of *K*. *pneumonia-*infected mice *via* producing TNF-α that markedly up-regulates the level of CCL20 in the lung epithelial cells (21). It is well-known that NCR^+^ ILC3s primarily express IL-22, but NCR^−^ ILC3s predominantly produce IL-17 (12). Consequently, it deserves to be investigated how NCR^−^ ILC3s home to the lungs, and whether pulmonary CD103^+^CD11b^+^ DCs function in lung-selective migration of NCR^+^ ILC3s.

Above all, ILC3s play a pivotal and previously underappreciated role in pulmonary TB immunity. They mainly mediate body's early defense against TB infection through the IL-17/IL-22 axis in a CXCL13-CXCR5-dependent manner with formation of iBALT, which bridges a gap between innate immune cells and adaptive immune responses and advances our understanding of pathogenesis of TB. During *Mtb* infection, stromal cells, FDCs, and B cells express CXCL13 in iBALT, by which circulating CXCR5-expressing ILC3s are attracted into the lungs to mediate protective immunity from *Mbt* (18). The breakthrough discovery not only opens a door to research on ILCs for their regulating TB infection, but also paves a way to develop a potential treatment regimen for TB or especially drug-resistant TB. We reasonably infer that this early protective effect of ILC3s would appear in intestinal *Mtb* infection. However, how to target ILC3s or their secreted functional molecules, which contributes to the body to resist *Mtb* invasion, needs further exploration. Besides, BCG vaccination in animals unexpectedly initiates a rapidly strong recruitment of ILC3s that confer a protective immune response to *Mtb* in the lungs *via* the intranasal route administration, suggesting that there exist lung memory-like ILC3s (22). Hence, ILC3s play an important part in protective immunity against TB beyond our current recognition. We imagine that the memory-like ILC3s might be developed as potential vaccines in the future.

## Author Contributions

FX conceived of this commentary. BZ and FX drafted it. CD was responsible for the critical reading. FX and RX revised and finalized the manuscript. All the authors read and approved the final manuscript.

## Conflict of Interest

The authors declare that the research was conducted in the absence of any commercial or financial relationships that could be construed as a potential conflict of interest.
